# Tisotumab vedotin–associated Stevens–Johnson syndrome in a patient with recurrent vulvar cancer: Vulnerability and antibody drug conjugate toxicity considerations

**DOI:** 10.1016/j.gore.2026.102065

**Published:** 2026-03-22

**Authors:** Isabel Chess, Ann Marie Mercier, Sarah Gross, Stephanie V. Blank, Monica Prasad Hayes

**Affiliations:** a Icahn School of Medicine at Mount Sinai, USA; b Department of Obstetrics, Gynecology, and Reproductive Science, Division of Gynecologic Oncology, Icahn School of Medicine at Mount Sinai, USA; c Department of Obstetrics, Gynecology, and Reproductive Science, Icahn School of Medicine at Mount Sinai, USA

**Keywords:** Recurrent vulvar cancer, Stevens Johnson Syndrome, Toxic Epidermal Necrolysis, Tisotumabvedotin, Adverse drug event, Antibody drug conjugate

## Abstract

**Background:**

Severe adverse events from novel cancer therapies are significant concerns, particularly in patients with recurrent disease who have limited treatment options and reduced reserve. We present a rare case of Stevens Johnson Syndrome (SJS) after initiation of tisotumab vedotin (TV) in an elderly patient with recurrent vulvar squamous cell carcinoma (SCC).

**Case:**

A 76-year-old female with recurrent poorly differentiated vulvar SCC presented with fever, painful rash, and altered mental status 11 days after starting TV. Medical history is notable for HIV (undetectable viral load on antiretroviral medication). Examination revealed a desquamating rash in the bilateral axillae and groin. She was admitted for urosepsis secondary to an obstructing right kidney stone. Two days later, her rash progressed with a positive Nikolsky sign and lesions found in the mouth, axillae, and back. Histopathology frozen section demonstrated SJS and her clinical picture was consistent with toxic epidermal necrolysis (TEN) with a mortality risk of > 90%. The patient and family elected comfort measures. She died on hospital day four.

**Conclusion:**

A phase III trial on patients with recurrent cervical cancer reported SJS in three patients (representing 1.2% of patients on TV), with one fatal outcome. Given that the median age was 51 and frailer patients were excluded, this trial may underestimate toxicity in a broader population. Multidisciplinary management is essential for frail patients with comorbidities receiving medications such as TV. Further research is necessary to improve outcomes of patients who suffer SJS/TEN and other serious adverse events after TV as its use expands.

## Highlights

• Stevens Johnson Syndrome (SJS) after tisotumab vedotin use is a rare adverse event.
• Older age and prior health conditions may increase the incidence of SJS.
• HIV is not a contraindication to antibody drug conjugates, but close monitoring is required.

## 1 Introduction

Recurrent vulvar squamous cell carcinoma (SCC) presents a significant therapeutic challenge as treatment outcomes vary depending on site and extent of disease recurrence.

Additionally, current treatment modalities are extrapolated from cervical and anal SCC data. For isolated nodal or distant recurrences, the National Comprehensive Cancer Network (NCCN) recommends systemic therapy with cisplatin/paclitaxel and carboplatin/paclitaxel, with or without bevacizumab, based on phase III cervical cancer trials. Second line treatments include single-agent paclitaxel or immunotherapies like cemiplimab and pembrolizumab (Abu-Rustum et al., 2024). Despite advances in treatment modalities, survival for recurrent vulvar SCC remains approximately 12–15 months (Woelber et al., 2012, Han et al., 2013).

Tisotumab vedotin (TV), an antibody-drug conjugate that targets tissue factor, represents an emerging therapeutic option for recurrent vulvar SCC. It received accelerated approval in 2024 from the US Food and Drug Administration (FDA) for the treatment of patients with recurrent and metastatic cervical cancer (DailyMed - TIVDAK- tisotumab vedotin injection, powder, for solution, 2026). While TV is not currently FDA approved for the treatment of vulvar SCC, it is recommended by the NCCN for management of recurrent vulvar SCC based on this data and the shared histologies and tissue factor expression across gynecologic squamous cell carcinomas.

The innovaTV trial established superior efficacy in recurrent cervical SCC when comparing TV to investigator’s choice chemotherapy (Vergote et al., 2024). TV was found to significantly extend median overall survival (11.5 months versus 9.5 months in the chemotherapy cohort). The safety profile with TV was generally reassuring, with the most common adverse events including alopecia (38%), epistaxis (30%), nausea (27%), conjunctivitis (26%), and fatigue (26%). Ocular toxicity was a unique concern with this drug, occurring in approximately 26% of patients. As such, close ophthalmologic examination and three sets of eye drops are used throughout the treatment. In this trial, there was one fatality attributed to Stevens-Johnson Syndrome (SJS) (incidence of 0.4%) (Vergote et al., 2024). Two additional patients discontinued TV due to SJS (Vergote et al., 2024). There are no reported cases of TEN reported in the literature. A recent study of adverse events across the FDA Adverse Event Reporting System (FEARS) database found that SJS was a new safety signal associated with TV, and had a reporting odds ratio of 9.91 (Li et al., 2025).

Given the increasing application of TV for treatment of gynecologic malignancies, early diagnosis and treatment of rare yet potentially fatal adverse effects is essential. In this case report, we present a fatal case of TEN, the most severe version of SJS, occurring after initiation of TV in a patient with recurrent vulvar SCC. We aim to highlight the importance of early identification and management of severe dermatologic toxicity associated with this novel targeted treatment.

## 2 Case report

A 76 year old female, BMI 27.9 kg/m^2^, ECOG functional status 2 with well-controlled HIV on HAART with an undetectable viral load, was diagnosed with stage IIIB poorly differentiated vulvar SCC. She was treated surgically with radical vulvectomy and bilateral inguinal lymph node dissection in October 2021. Surgical pathology demonstrated a sentinel lymph node and superficial inguinal lymph nodes positive for malignancy. She completed four cycles of cisplatin chemotherapy with pelvic radiation (total dose 5600 cGy) in February 2022. The patient was then lost to followup.

She returned in October 2024 due to a new induration on the anterior vulva, at which time Positron Emission Tomography (PET) scan demonstrated fluorodeoxyglucose (FDG) avid nodules in the lungs and FDG avid supraclavicular and hilar lymph nodes consistent with recurrent disease. She subsequently underwent six cycles of chemotherapy with carboplatin and paclitaxel, completed in April 2025. A post-treatment PET scan demonstrated progressive disease, with increases in nodal size, number and FDG activity. She was started on TV for progression of disease in June 2025.

**Fig. 1:**
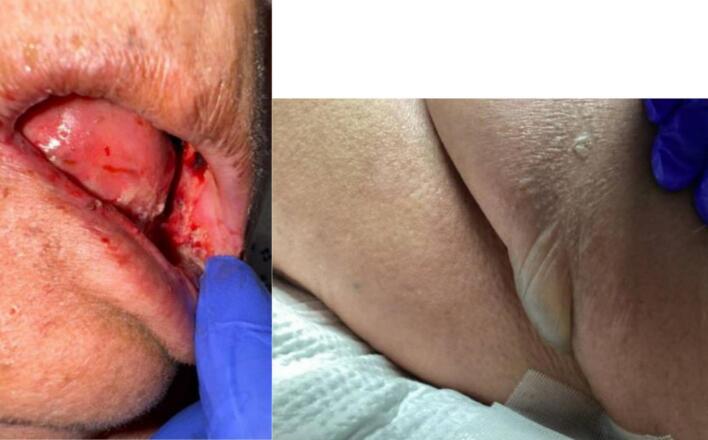
Representative clinical findings. Oral mucosa shows hemorrhagic erosions and dry membranes.

Eleven days after initiating TV, she presented to the ED for a desquamating and painful rash, oral lesions, fevers, nausea, vomiting, cough, altered mental status, and visual hallucinations for four days. On arrival to the ED, the patient was found to be febrile to 100.5F and hypotensive to 90 s/50 s with a lactate of 5.4. Physical exam was notable for generalized abdominal tenderness, oral thrush, and a desquamating rash in bilateral axilla and groin (photos below in Fig. 1). Vulvar exam showed no discrete lesions and no bleeding. Notable data and labs on admission included, potassium 6.5 mEq/L, lactate 3.5 mmol/L, WBC 0.5 cells/µL with an unmeasurable absolute neutrophil count (ANC), platelets 180 cells/µL, creatinine 2.8 mg/dL (from baseline of 1.0), and BUN 34 mg/dL. A respiratory viral panel was sent and was negative. The sepsis pathway was activated. Computed Tomography Angiography (CTA) imaging of the chest, abdomen and pelvis showed large impacted right kidney stones associated with hydronephrosis, concerning for urosepsis and multifocal pneumonia.

The patient was admitted for management of presumed urosepsis, acute kidney injury, and pneumonia. She was started on cefepime and pancultured. Interventional radiology placed a right percutaneous nephrostomy (PCN), and several hours later, the patient became hypotensive (80 s/40 s), tachycardic (108) and febrile (100.1F). She was given IV fluids and acetaminophen. Despite fluid resuscitation, the patient continued to be hypotensive (60 s/40 s) and triggered the Systemic Inflammatory Response Syndrome (SIRS) management pathway. She was given additional IV fluids and one dose of vancomycin. A rapid response team was called. Labs were significant for lactate of 3.1 mmol/L, WBC of 0.5 cells/µL, Calcium of 5.9 mg/dL and Creatinine of 2.8 mg/dL. Calcium gluconate and albumin were given, and another infectious workup was collected.

**Fig. 2:**
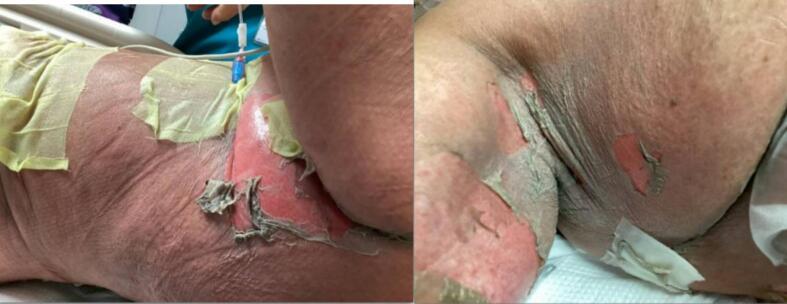
Widespread epidermal detachment and necrolysis with sloughing of skin in axilla.

At this time, physical exam was remarkable for a positive Nikolsky sign − a clinical sign where pressure on the skin causes the top layers to separate and peel, indicating a loss of skin cell adhesion. Lesions were found in the mouth, under arms, and along the back (Fig. 2). Dermatology was consulted due to concerns for SJS. Skin biopsy was performed and confirmed SJS on frozen section with a Score of Toxic Epidermal Necrolysis (SCORTEN) 5. This assessment corresponds to > 90% mortality. Given the skin reaction encompassed more than 30% of the patient’s body surface area (BSA), the patient was diagnosed with Toxic Epidermal Necrolysis (TEN). The decision was made to transfer her to the intensive care unit (ICU) given the critically ill state. She was given etanercept. The patient remained persistently hypotensive on vasopressors overnight. A goals of care meeting was held with the family and the decision was made to pursue comfort measures and not transfer to the burn unit. The patient died later that day on hospital day 4.

## 3 Discussion

SJS is a rare and life-threatening mucocutaneous adverse reaction that can occur for a myriad of reasons including adverse drug reactions. TEN is a severe form of SJS when the skin reaction encompasses more than 30% of the patient’s BSA. To our knowledge, no published case reports or clinical trial data document SJS after administration of TV in patients with recurrent vulvar SCC. This patient had risk factors for adverse drug events, including advanced age, frailty, and HIV infection. Of note, SJS secondary to inpatient antibiotic exposure is unlikely in this case given the typical 4 to 28 day onset from the delayed, immune-mediated hypersensitivity reaction in SJS (Lee et al., 2023, Duong et al., 2017).

HIV infection is not a contraindication to TV use or other antibody drug conjugate therapies (ADC); however, data regarding ADC use in this population remain limited (Levinson et al., 2016). The NCCN recommends close coordination between oncologists and HIV specialists to monitor for potential drug-drug interactions and hematologic toxicity (Guidelines Detail. NCCN., 2026). While this patient had an undetectable viral load, caution is warranted when initiating TV in individuals with baseline cytopenias or advanced immunosuppression (CD4 count < 200 cells/µL). In patients with preexisting cytopenias, prophylactic myeloid growth factor support (eg, pegfilgrastim) should be considered given the risk of treatment-related leukopenia. Additionally, antiretroviral regimens containing ritonavir or cobicistat may require modification, as these CYP3A4 inhibitors could alter the metabolism of TV and increase toxicity risk (Guidelines Detail. NCCN., 2026).

One study evaluating the FDA adverse event reporting data for multiple cancer treatment modalities found that patients who were older than 65, female, or had previous severe cutaneous reactions were more likely to develop SJS (Liu et al., 2025). This study suggests that an age greater than 65 years may be a risk factor for the development of SJS when using anti-cancer drugs. This has implications for this case report, as patients who are older may be more at risk for serious adverse events. Additionally, patients with prior history of SJS or severe cutaneous reactions were excluded from the clinical trials for TV, underscoring the need for careful patient selection and monitoring.

In addition, patients included in the InnovaTV trial had functional status of ECOG 0–1. The patient in this report had a functional status of ECOG 2. This patient had a modified frailty index (mFI-5) score of 2 (for functional dependence and hypertension on medication). Recent studies in gynecologic oncology surgery have found that higher frailty, with screening using mFI-5, was associated with adverse postoperative events and chemotherapy complications (Mah et al., 2022). Currently no evidence exists in regards to the impact of frailty on outcomes of oncology patients treated with antibody drug conjugates. However, the lack of representation of elderly and frail patients in InnovaTV and other clinical trials on antibody drug conjugates presents a possible unrecognized source of increased adverse events in these populations. This underscores that trial populations are not always “real world” populations, and that starting dose adjustments for individual patients not meeting every inclusion criterion for trial may be necessary. Further research is needed to assess the impact of frailty on patient outcomes and adverse events when prescribing TV.

While severe cutaneous adverse reactions, including SJS/TENs, occur across multiple ADCs, the incidence varies by agent, with enfortumab vedotin demonstrating the highest risk (15 fold increased risk compared to other drugs) and carrying a boxed warning for these reactions (Yang et al., 2021, Lacouture et al., 2022). Differences in antibody targets may explain varying incidence rates between these agents, with TV targeting tissue factor and enfortumab vedotin targeting nectin-4, which is highly expressed in epidermal keratinocytes (Lacouture et al., 2022). Further exploration of the mechanism of ADC mediated severe cutaneous reactions may further explain the differences seen in rates of SJS.

Our case demonstrates the need for vigilant monitoring for early signs of SJS and prompt intervention to mitigate morbidity and mortality. HIV infection alone should not preclude the use of antibody–drug conjugates, but careful multidisciplinary management is essential to mitigate hematologic toxicity and drug–drug interactions. Additionally, newer evidence suggesting that older age may increase the incidence of SJS for patients on cancer therapies brings up interesting questions on how to appropriately risk stratify in our gynecologic cancer patient population. Further research is necessary to clarify the risk factors that put patients at heightened risk for adverse cutaneous reactions as TV is given to more cancer patients.

## 4 Consent statement

Informed consent was obtained for publication of this case report and accompanying images.

## CRediT authorship contribution statement

**Isabel Chess:** Writing – review & editing, Writing – original draft, Formal analysis, Conceptualization. **Ann Marie Mercier:** Writing – review & editing, Formal analysis, Conceptualization. **Sarah Gross:** Writing – review & editing, Formal analysis. **Stephanie V. Blank:** Writing – review & editing, Conceptualization. **Monica Prasad Hayes:** Writing – review & editing, Conceptualization.

## Declaration of competing interest

The authors declare that they have no known competing financial interests or personal relationships that could have appeared to influence the work reported in this paper.
